# Dual task effect on postural control in patients with degenerative cerebellar disorders

**DOI:** 10.1186/s40673-015-0025-z

**Published:** 2015-05-08

**Authors:** Heike Jacobi, Juliane Alfes, Martina Minnerop, Jürgen Konczak, Thomas Klockgether, Dagmar Timmann

**Affiliations:** Department of Neurology, University Hospital of Bonn, Bonn, Germany; Department of Neurology, University Hospital of Essen, University of Duisburg-Essen, Essen, Germany; Department of Surgery, Knappschaftskrankenhaus Recklinghausen, Recklinghausen, Germany; Institute of Neuroscience and Medicine (INM-1), Research Centre Juelich, Juelich, Germany; School of Kinesiology, Center for Clinical Movement Science, University of Minnesota, Minnesota, USA; German Center for Neurodgenerative Disorders (DZNE), Bonn, Germany

**Keywords:** Cerebellar ataxia, Dual task, Postural control, Static and dynamic posturography

## Abstract

**Background:**

The cerebellum plays an important role for balance control and the coordination of voluntary movements. Beyond that there is growing evidence that the cerebellum is also involved in cognitive functions. How ataxic motor symptoms are influenced by simultaneous performance of a cognitive task, however, has rarely been assessed and some of the findings are contradictory. We assessed stance in 20 patients with adult onset degenerative almost purely cerebellar disorders and 20 healthy controls during single and dual task conditions (verbal working memory task). To objectively measure postural sway and the impact of somatosensory, visual and vestibular inputs we used static and dynamic posturography with the Sensory Organization Test (SOT).

**Results:**

In both groups, cerebellar patients and controls, dual tasking reduced all sway parameters. Reduction of sway path was higher in cerebellar patients and increased with the difficulty of the postural task. The frequency of falls was higher in the patients group especially during the more challenging conditions and dual task performance in particular increased the risk of falls in cerebellar patients.

**Conclusion:**

Dual task conditions had a larger impact on sway parameters in patients with chronic cerebellar disorders than in healthy controls and lead to an increased risk of falls. As performing two tasks simultaneously is common and therefore important in daily life dual task exercises should be part of physical therapy programs for cerebellar patients.

## Background

The cerebellum plays an important role for balance control and the coordination of voluntary movements. Cerebellar degeneration leads to ataxic symptoms, such as ataxia of stance, gait and limbs. Beyond that there is growing evidence that the cerebellum is also involved in cognitive functions [1-3], particularly executive control including working memory and language, and possibly visuospatial function [1]. Cerebellar involvement in cognition is supported by human cerebellar lesion studies and functional brain imaging studies in healthy subjects [4-7].

A deterioration of motor performance especially gait control and a higher risk of falls during simultaneous performance of a cognitive task has been shown for older adults and several neurological diseases, such as Parkinson’s or Alzheimer’s disease [8-13]. There is only little information on the influence of dual task performance on motor execution in cerebellar patients.

An interaction of cognitive and motor functions of the cerebellum is quite conceivable. A recent functional MRI study in healthy subjects showed that during simultaneous performance of cognitive and motor tasks areas in the cerebellar vermis and anterior lobe were additionally activated and had functional connectivity with extensive motor- and cognitive-related regions [14]. So, these regions seem to play an important role to adjust and integrate motor and cognitive networks.

Performing two tasks simultaneously is common in daily life and therefore of high relevance for cerebellar patients. How ataxic motor symptoms are influenced by simultaneous performance of a cognitive task, however, has rarely been assessed, and some of the findings are contradictory. Lang and Bastian [15] examined cerebellar patients and controls in an upright posture while performing a figure-8-movement at the same time as a working memory task. They showed that cognitive and motor performance interferes in patients with cerebellar disorders, and dual tasking worsened motor performance in cerebellar subjects to pre-practice levels. They also provided evidence that the cerebellum plays a role for shifting movement performance from an attentionally demanding to a more automatic state. Ilg and colleagues [16] examined the interaction of working memory and different gait tasks in patients with focal cerebellar lesions. They found decreased performance of working memory and increased tandem gait variability during dual task conditions. By contrast, Salih and colleagues [17] reported an improvement of cerebellar ataxia in a patient with a chronic lesion of the right dentate nucleus when climbing a 10-step staircase and simultaneously performing cognitive tasks (word generation or arithmetic tasks) whereas the same patient needed more time to walk a 10 meter distance when performing a cognitive task. The authors hypothesized that potentially interferences between motor and cognitive cerebello-thalamo-cortical loops can be influenced by specific cognitive tasks and improve ataxic symptoms.

To analyze how cognitive distraction affects stance in patients with chronic degenerative cerebellar disorders, we assessed different stance conditions during single and dual task conditions in cerebellar patients and healthy controls.

Parts of this study have been published as medical doctoral thesis of one of the authors [18].

## Results

### Sensory Organization Test (SOT)

**Figure 1:**
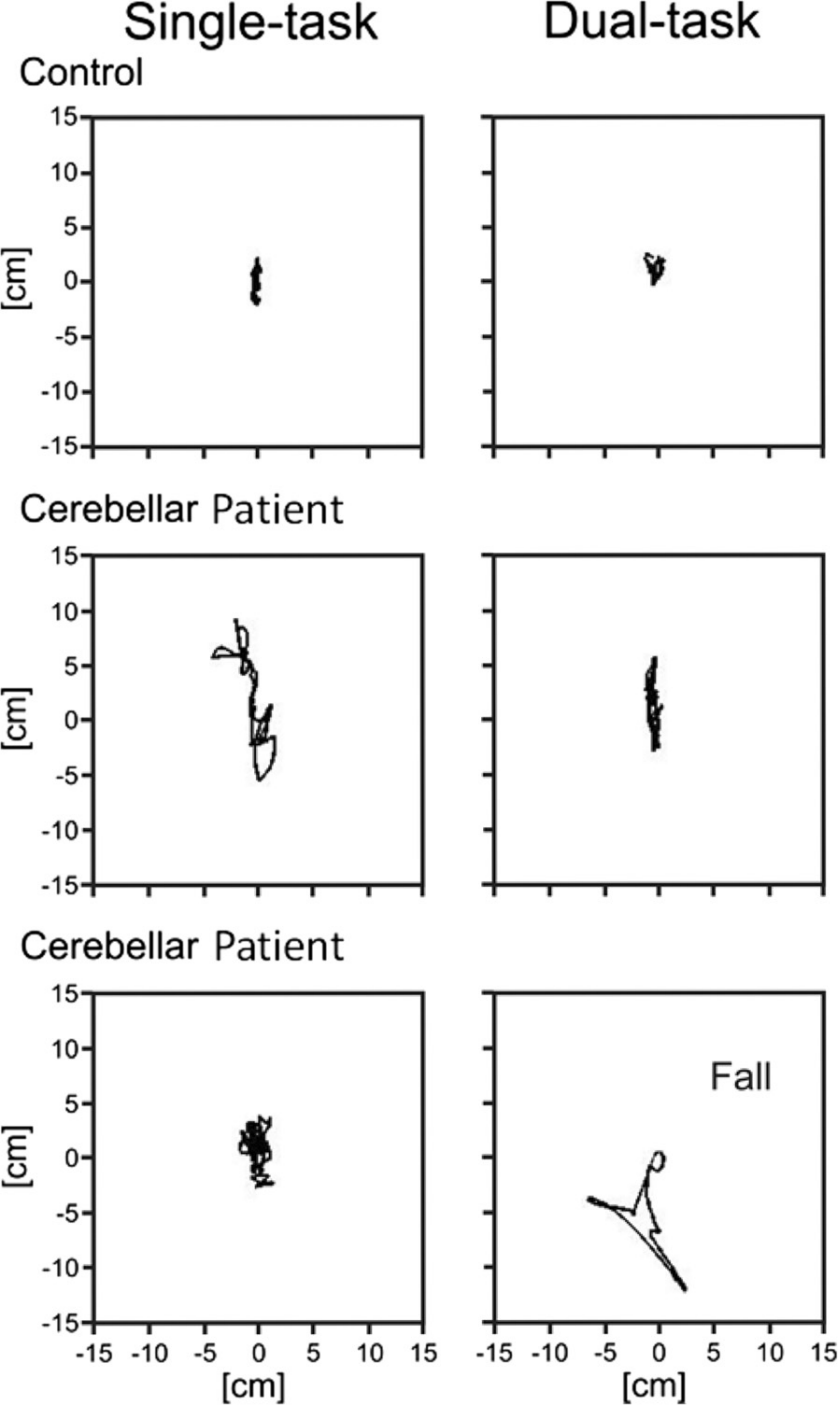
Sway path of center of gravity in condition 6 comparing single and dual task conditions for a healthy control and two cerebellar patients; modified according to [18].

Typical examples of sway path of a healthy control participant and two cerebellar patients with and without dual tasking during condition 6 are shown in Figure 1. Sway path of the control participant is short with an emphasis on anteroposterior sway during single task and slightly reduced during dual task. Anteroposterior and mediolateral sway path of the cerebellar patient is increased compared to the control during both conditions, single and dual task, and decreases during dual tasking. The last example shows sway path of a cerebellar patient before a fall with increased sway in anteroposterior and mediolateral direction during dual task.

**Figure 2:**
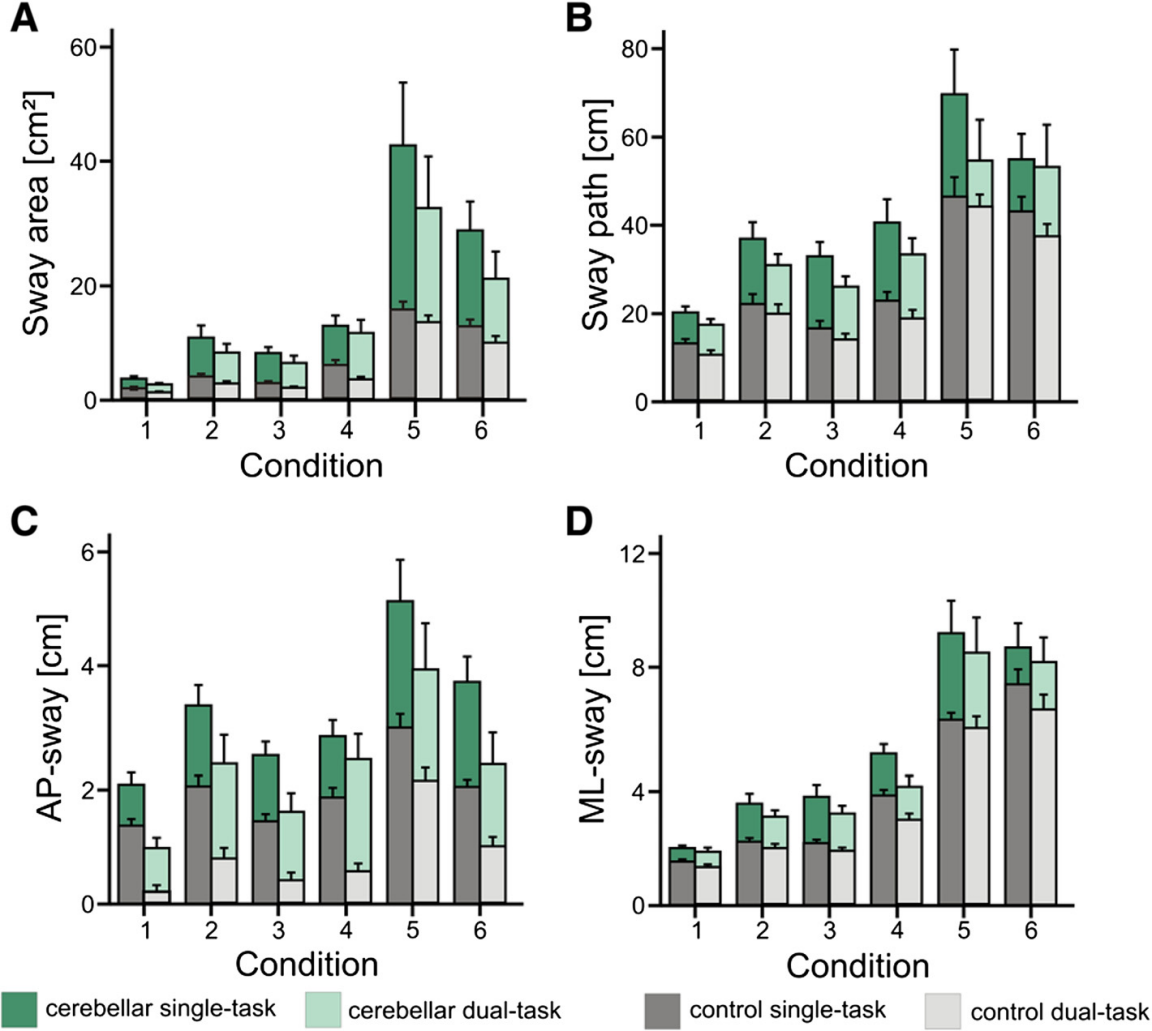
Sway parameters of patients and controls during single and dual task for all stance conditions (given as mean values with standard deviation); modified according to [18]. Sway parameters of cerebellar patients are marked green (dark green single and light green dual task), of controls grey (dark grey single and light grey dual task).

Cerebellar patients showed higher sway area (p = 0.002) and anteroposterior sway (p = 0.001) than controls [significant effect for group; 2 Groups (controls vs. cerebellar) x 2 Sets (single vs. dual task) x 6 Sway Conditions (6 SOT conditions) ANOVA] (Figure 2; Table 1). Group differences were close to significance considering sway path (p = 0.057) and mediolateral sway (p = 0.057). In both groups sway area (p < 0.001), sway path (p < 0.001), anteroposterior (p < 0.001) and mediolateral sway (p < 0.001) differed between the six different conditions (effect of condition; Table 1) and tended to show highest values in condition 5 and 6 (Figure 2). For sway area (p = 0.005) but not for the other sway parameters, the difference between the conditions was higher in cerebellar patients than in healthy controls (significant group by condition interaction; Table 1).

**Table 1:** **Analysis of variance of sway measures**

**Parameter**	**Effect**	**degrees of freedom, f-value**	**p-value**
Sway area	Dual task effect	F(1;26) = 24.197	**<0.001**
	Dual Task effect*group	F(1;26) = 0.028	0.868
	Condition	F(1.42;36.932) = 70.9	**<0.001**
	Condition*group	F(1.42;36.932) = 7.46	**0.005**
	Dual task effect*condition	F(2.547;66.232) = 1.559	0.213
	Dual task effect* condition*group	F(2.547;66.232) = 0.618	0.580
	Group	F(1;26) = 11.192	**0.002**
Sway path	Dual task effect	F(1;26) = 49.173	**<0.001**
	Dual task effect*group	F(1;26) = 0.569	0.457
	Condition	F(2.219;57.69) = 72.382	**<0.001**
	Condition*group	F(2.219;57.69) = 0.649	0.542
	Dual task effect*condition	F(3.27;85.033) = 2.382	0.070
	Dual task effect* condition*group	F(3.27;85.033) = 4.127	**0.007**
	Group	F(1;26) = 3.958	0.057
Anteroposterior sway	Dual task effect	F(1;26) = 16.024	**<0.001**
	Dual task effect*group	F(1;26) = 0.09	0.926
	Condition	F(2.6;67.59) = 52.9	**< 0.001**
	Condition*group	F(2.6;67.59) = 3.242	0.033
	Dual task effect*condition	F(5;130) = 1.471	0.204
	Dual task effect* condition*group	F(5;130) = 0.647	0.664
	Group	F(1;26) = 15.704	**0.001**
Mediolateral sway	Dual task effect	F(1;26) = 11.925	**0.002**
	Dual task effect*group	F(1;26) = 0.715	0.406
	Condition	F(2.113;54.954) = 125.941	**< 0.001**
	Condition*group	F(2.113;54.945) = 1.341	0.271
	Dual task effect*condition	F(3.124;81.233) = 2.82	0.042
	Dual task effect* condition*group	F(3.124;81.233) = 1.908	0.132
	Group	F(1;26) = 3.953	0.057

2 Groups (controls vs. cerebellar) x 2 Sets (single vs. dual task) x 6 Sway Conditions (6 SOT conditions) ANOVA.

p-values < 0.0125 were considered significant (Bonferroni corrected). Modified according to [18].

Double or triple interaction effects between the variables are marked with a *.

In both groups, cerebellar patients and controls, dual task significantly reduced sway area (p < 0.001), sway path (p < 0.001), anteroposterior sway (p < 0.001) and mediolateral sway (p = 0.002) compared to the single task (Table 1; Figure 2). Effects of dual task did not differ between the different conditions (dual task by condition interaction) (Table 1, Figure 2).

Overall the reduction did not differ between the cerebellar and the control group (no significant dual task by group interaction). Taking into account the different conditions, we found that cerebellar patients showed significantly less sway path during dual task during the more advanced conditions (p = 0.007; group by dual task by condition interaction), this interaction was not significant for sway area, anteroposterior and mediolateral sway (Table 1).

**Figure 3:**
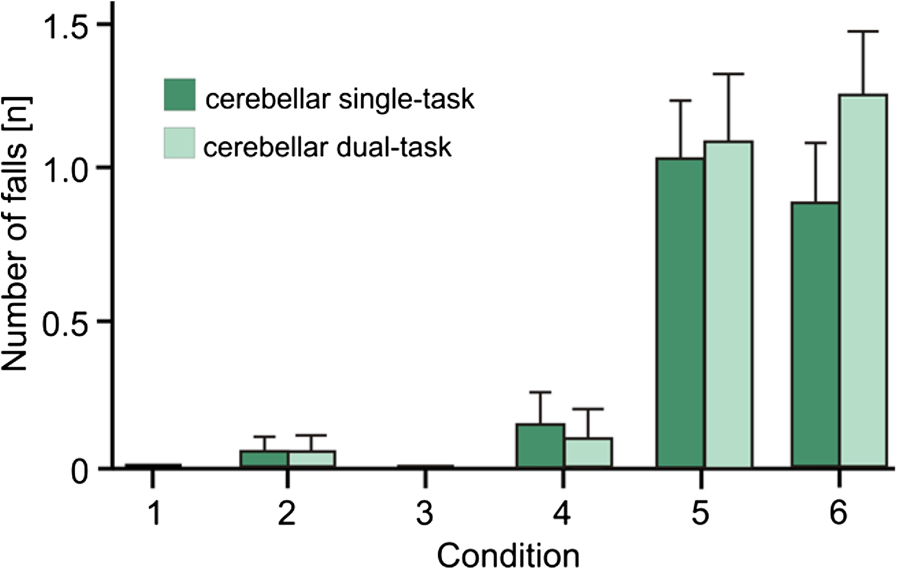
Number of falls of patients during single and dual task conditions for all stance conditions (given as mean values with standard deviation); modified according to [18]. Number of falls of cerebellar patients is marked green (dark green single and light green dual task).

The number of falls in all trials and conditions were counted per patient. Frequency of falls was different between conditions (p < 0.001; effect of condition; 2 Groups (controls vs. cerebellar) x 2 Sets (single vs. dual task) x 6 Sway Conditions (6 SOT conditions) ANOVA) and significantly higher in the patients’ group (p < 0.001; group effect): While there was only one fall in condition 5 in the control group, falls were frequent in the patient group (43 falls during single task, 50 falls during dual task conditions), mainly during conditions 5 and 6 (86 of 93 falls) (Figure 3). In cerebellar patients dual tasking also increased the risk of falls with increasing difficulty of the conditions (p < 0.001; group by dual task by condition interaction).

**Figure 4:**
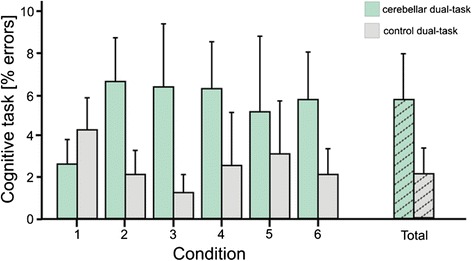
Percentage error in the cognitive task during dual task for all stance conditions (given as mean values with standard deviation); modified according to [18]. Percentage error of cerebellar patients is marked green, of controls grey.

Errors in the cognitive task were calculated as percentage error of all target letters to be counted. The mean error was numerically higher in the patient group (75 vs. 39 miscounted letters in patients vs. controls), but this difference was not significant (p = 0.105; 2 Groups (controls vs. cerebellar) x 2 Sets (single vs. dual task) x 6 Sway Conditions (6 SOT conditions) ANOVA) (Figure 4). However, because falls of cerebellar patients during condition 5 and 6 were so frequent and patients stopped counting, these trials had to be excluded from this analysis. Hence error ratio in the patient group actually underestimates the effect of the dual task on memory error.

## Discussion

In this study we assessed stance in patients with chronic cerebellar disorders and healthy controls during single and dual task conditions to see how a simultaneous cognitive task affects balance. In both groups, cerebellar patients and controls, dual tasking reduced all sway parameters. Reduction of sway path was higher in cerebellar patients and increased with the difficulty of the postural task. The frequency of falls was higher in the patients group especially during the more advanced conditions. In cerebellar patients dual task performance increased the risk of falls.

As expected, cerebellar patients showed ataxic stance with higher sway parameters than controls in all observed conditions. Deficits became most apparent in condition 5 and 6, those conditions in which platform tilt and/or vision were sway-referenced. Therefore quality of stance mainly depends on the vestibular or vestibulocerebellar system. This is in line with the results of Gatev and colleagues [19] who assessed 25 patients with cortical cerebellar atrophy (CCA), nine with olivo-ponto-cerebellar atrophy (OPCA) and 10 healthy controls with the Sensory Organization Test. They reported more body sway as well as higher frequency of falls in the patients’ group and in general more sway during condition 5 and 6.

The main aim of our study was to examine how cognitive distraction affected motor performance in patients with chronic cerebellar disorders. In both groups, cerebellar patients and controls, dual task reduced all sway parameters. Taking into account the different conditions, we found that cerebellar patients showed a significant reduction of sway path during dual task in the more advanced conditions. On the other hand dual task increased the risk of falls during the more advanced conditions. Decrease of sway might be due to higher co-contractions of antagonistic muscle groups during postural adjustments to compensate ataxia [20]. Cerebellar patients often show increased muscle stiffness [21]. This is may also reflect a primary cerebellar deficit. Increased co-contraction is supposed to decrease the ability to react to challenging balance leading to an increased risk of falls. In fact, physiotherapy in cerebellar patients aims at decreasing co-contractions and compensatory stiffening of limbs. The efficacy of such physiotherapeutic interventions that train dynamic stability of stance and gait has recently been shown [22,23].

Our observation that dual task worsened motor performance in cerebellar subjects is in line with the results of Lang and Bastian [15] who examined cerebellar patients and controls with an upper limb motor task and Ilg and colleagues [16] who examined the gait of cerebellar patients and controls under dual task conditions. Our results are at variance with findings of Salih et al. [17] who found a partial improvement of ataxic symptoms during the concurrent performance of a cognitive task. One may argue that decreased sway under dual task conditions reflects an improvement of postural parameters in cerebellar patients in the present study. However, as discussed above, this may also be interpreted as a sign of malcompensation leading to the observed increased number of falls.

A deterioration of motor performance during dual task conditions has been shown for older adults and other neurological conditions, such as Parkinson’s or Alzheimer’s disease [10-13]. Furthermore combining physical and cognitive training has been shown to be more beneficial in improving gait stability and preventing falls in older adults [8,9]. Performing a motor and a cognitive task simultaneously potentially diminishes the attention available for stance control. This may partly explain the changes of sway parameters in both, cerebellar patients and healthy controls. Changes, however, were more prominent in the more difficult conditions in cerebellar patients suggesting an additional specific cerebellar effect on motor performance during dual tasking. There is increasing evidence that the cerebellum not only plays an important role in motor control but is also involved in cognitive processes [1,24-27]. In particular, it has been shown that the cerebellum plays a role in verbal working memory [28,29]. More specifically, a recent functional MRT study suggested that the cerebellum is directly involved in dual task performance by adjusting and integrating motor and cognitive networks [14].

Errors in the working memory task were numerically higher in the patients’ group, but this difference was not significant. This does not contradict the literature, because overall, verbal working memory deficits tend to be mild in adult patients with chronic cerebellar disorders [25]. However, error ratio in the patients group might have been misleadingly low, because falls of cerebellar patients were frequent, especially during the more advanced conditions and patients stopped counting the letters, so these trials had to be excluded from the analysis. Likewise, we cannot exclude that errors increased in the more advanced postural conditions. Furthermore, working memory task had not been assessed while sitting or lying, and the effect of unperturbed stance on cognitive performance could not be assessed. A recent dual task study in cerebellar patients reported an influence of motor task complexity on the performance in a working memory task [16].

## Conclusion

In summary sway parameters during dual task conditions were changed in patients with chronic cerebellar disorders and lead to an increased risk of falls. As performing two tasks simultaneously is common and therefore important in daily life dual task exercises should be part of physical therapy programs for cerebellar patients.

## Methods

### Participants

A group of 20 patients with adult onset degenerative almost purely cerebellar disorders [sporadic adult onset ataxia (SAOA): 12; spinocerebellar ataxia type 6 (SCA6): 3; auto-immune cerebellar ataxia with GAD-antibodies: 2; purely cerebellar autosomal dominant ataxia (ADCA-III): 2; cerebellitis: 1] and 20 age-matched healthy controls [58.4 ± 11.64 years vs 58.25 ± 11.66 years (mean age ± SD)] were included in the study. Both groups consisted of 10 female and 10 male persons. Severity of ataxia was minor to moderate [ICARS 26.7 ± 11.2; SARA-score 10.2 ± 4.4 (mean ± SD)].

Participants were consecutively recruited within a period between March and July 2010. Inclusion criteria were progressive, almost purely cerebellar ataxia (patients) and age between 18 and 75 years (both groups). Exclusion criteria were other neurological diseases or symptoms other than cerebellar (patients), neurological or orthopaedic diseases (healthy controls) and the intake of sedatives (excluding low-dose treatment of antidepressants).

### Ethics, consent and permissions

All procedures performed in studies involving human participants were in accordance with the ethical standards of the institutional research committee and with the 1964 Helsinki declaration and its later amendments or comparable ethical standards. The study has approved by the local ethics committee (09–4170). Informed consent was obtained from all individual participants included in the study.

### Assessment instruments

The severity of ataxia was assessed with the Scale for the Assessment and Rating of Ataxia (SARA), an 8-item clinical rating scale ranging from 0 (no ataxia) to 40 (most severe ataxia) [30] and the International Cooperative Ataxia Rating Scale (ICARS), a 19-items clinical rating scale ranging from 0 (no ataxia) to 100 (most severe ataxia) [31]. The investigator (DT) was trained in the application of the applied scales.

Static and dynamic posturography was performed with the Sensory Organization Test (SOT) using the EquiTest system (neuroCom, Inc., USA) to objectively measure postural sway and the impact of somatosensory, visual and vestibular inputs under six different visual and support-surface conditions: 1. Eyes open, fixed platform surface and background, 2. Eyes closed, fixed platform surface and background, 3. Eyes open, fixed platform surface and sway-referenced visual background, 4. Eyes open and sway-referenced surface, 5. Eyes closed and sway-referenced surface and 6. Eyes open, sway-referenced surface and visual background. Stance width was fixed in all subjects. Ground reaction forces were recorded for 20 seconds with a sampling rate of 100 Hertz. The SOT consisted of 18 trials in total, 3 trials per postural condition. The postural test system recorded data of the four force transducers and the associated shear forces between the two force plates. With customized software based on MATLAB technical programming language, the raw data were calibrated and subsequently filtered offline using a 4th-order lowpass Butterworth filter with a cut-off frequency of 3 Hz. Based on the filtered force-time data of each 20 s trial, the following postural variables were computed: Sway path length defined as the total length of the path participant’s centre of gravity, sway area as area between the maximum excursions of a participant’s centre of gravity along the mediolateral (ML) and anterioposterior (AP) axes. Furthermore, the range of sway in the AP and ML direction was calculated.

All six stance conditions were tested in a single and dual task paradigm. In the single task treatment, participants performed the standard SOT test, in the dual task paradigm, they performed an additional verbal working memory task. During the verbal memory task participants listened to a 20-letter-sequence at one Hz frequency, that consisted of a random series of four letters (A, G, M, O). Participants were asked to count the number of a target letter in every sequence. The sequence and target letter changed every trial. Percentage error was calculated with the maximum number of letters to be counted defined as 100 percent. Each participant completed two sets of the Sensory Organisation Test. During the first set, the first two trials per condition were performed without cognitive distraction (single task), the last of the three trials per condition under dual task conditions (condition 1: single task, single task, dual task; condition 2: single task, single task, dual task; etc.). During the second set the first trial per condition was performed without cognitive distraction (single task), the second trial under dual task conditions and the third trial again without cognitive distraction (single task) (condition 1: single task, dual task, single task; condition 2: single task, dual task, single task; etc.).

### Statistical analysis

For each of the six different conditions the first trial of the first set (single task) and the last trial of the second set (single task) were excluded from the final analysis. Therefore, four trials per condition (two under single and two under dual task conditions) were analysed and arithmetic means were compared. For each sway parameter (sway path, sway area, anteroposterior (AP) and mediolateral (ML) sway) a 2 Groups x 2 Sets x 6 Sway Conditions repeated measures analysis of variance (ANOVA) with Bonferroni correction was performed. P values < 0.0125 were considered significant (that is 0.05 divided by four, the number of ANOVAs). Falls were excluded from the analysis of sway parameters and analysed separately with an analysis of variance (ANOVA). Analyses were performed with IBM SPSS 18.0 (SPSS Inc., 2009).
